# A unusual presentation of liver failure caused by Ibuprofen-sustained release capsules: A case report

**DOI:** 10.1097/MD.0000000000036997

**Published:** 2024-01-26

**Authors:** Yan Liu, Ming-wei Liu

**Affiliations:** a Department of Gastroenterology, The People’s Hospital of Lincang City, Lincang, Yunnan, China; b Department of Emergency Medicine, The First Affiliated Hospital of Kunming Medical University, Kunming, Yunnan, China; c Department of Emergency Medicine, The First Affiliated Hospital of Guizhou University of Traditional Chinese Medicine, Guiyang, Guizhou, China.

**Keywords:** case report, diagnosis, ibuprofen-sustained release capsules, liver failure, treatment

## Abstract

**Rationale:**

Previous studies have shown that acetaminophen has the potential to induce hepatotoxicity in patients, rendering it a prominent drug implicated in the development of acute hepatic failure. However, there is currently no available literature reporting the impact of ibuprofen-sustained release capsules on liver failure.

**Patient concerns:**

A 65-year-old man was presented with a 4-day history of tea-colored urine with oil avoidance, jaundiced skin, and anorexia, and impaired liver function. One ibuprofen-sustained release capsule was taken on the day before the onset of the disease due to “headache.”

**Diagnoses:**

A diagnosis of this patient was made of liver failure due to taking ibuprofen-sustained release capsules.

**Interventions:**

Initially, the patient discontinued the use of hepatotoxic drugs in order to prevent further exposure. Subsequently, the patient underwent a standard therapeutic regimen, which encompassed the administration of hepatoprotective agents, nutritional support drugs, correction of acid-base imbalances, and electrolyte abnormalities, as well as other relevant treatments.

**Outcomes:**

After 9 days of hepatoprotective and nutritional supplement therapy, the patient saw notable improvement in symptoms, reporting an absence of discomfort, subsided skin jaundice, clear urine, and liver function tests returning to a near normal range. The patient was granted permission to be discharged from the hospital while being prescribed drugs. After 2 weeks of follow-up, the patient reported an absence of discomfort and exhibited normal results in the liver function test.

**Conclusions:**

Liver failure caused by ibuprofen-sustained release capsules has not been reported. It is worth noting that conventional treatments such as suspending offending agents, and administration of hepatoprotective agents and nutritional support drugs have proven to be successful.

**Lesson:**

There is currently no known peer-reviewed literature indicating that the administration of ibuprofen-sustained release capsules leads to liver failure. When patients taking ibuprofen-sustained release capsules encounter symptoms such as anorexia, skin jaundice, lack of appetite, and nausea, it is recommended that they undertake a cardiac and liver function tests. In the event that ibuprofen-sustained release capsules induce liver injury, it is imperative to administer timely and immediate medical intervention.

## 1. Introduction

Drug-induced liver injury (DILI) is a condition characterized by hepatocyte toxicity resulting from the administration of medications and/or their metabolites. It is frequently encountered in clinic and its prevalence has been steadily rising over the years, ranking second only to viral hepatitis and hepatic steatosis.^[1]^ It is known that there are >1100 marketed drugs with potential hepatotoxicity in the world, including antiinfectious drugs (including antituberculosis drugs), antitumor drugs, nonsteroidal antiinflammatory drugs (NSAIDs), hormones, and herbal supplements.^[2]^ The statistical data indicate that an important percentage of drugs that have been identified as causing liver injury are over-the-counter drugs.^[3]^ These drugs have received limited attention in terms of the liver damage they might cause, leading to their characterization as “invisible killers.”^[4]^ NSAIDs mainly refer to a class of nonsteroidal drugs with antiinflammatory, analgesic, and antipyretic effects, which are the most widely used drugs in clinic. Long-term use can cause serious adverse side effects.^[5]^ Its gastrointestinal toxicity has been well-known by clinicians, and liver injury has gradually received attention. An increasing number of clinical cases indicate that NSAIDs have the potential to induce liver injury and exhibit hepatotoxicity.^[6]^ Clinicians have long been concerned about the contradiction between the effectiveness of NSAIDs and their potential for causing liver injury. The administration of this class of drugs has to be discontinued due to hepatotoxic effects, particularly in patients with preexisting liver conditions, resulting in a significant impact on the application of NSAIDs.

Previous studies have shown that acetaminophen has the potential to induce hepatotoxicity in patients, rendering it one of the primary drugs that cause acute liver failure.^[7]^ Liver failure caused by ibuprofen-sustained release capsules has not been reported so far. This article provides a comprehensive overview of a specific case involving liver failure induced by taking ibuprofen-sustained release capsules. The purpose of this study is to serve as a valuable resource for healthcare professionals in terms of both the treatment and diagnosis of liver failure resulting from ibuprofen usage.

## 2. Case report

### 2.1. Ethics approval and consent to participate

Informed written consent was obtained from the patient for the publication of this case report and the accompanying images.

This study was reviewed and approved by the local ethics committee of First Affiliated Hospital of Kunming Medical University. Procedures followed were in accordance with the Helsinki Declaration of 1975, as revised in 2000.

### 2.2. Medical history

The patient was a 65-year-old male who took 1 capsule of ibuprofen slow-release capsules for “headache” 4 days ago. The next day, he developed tea-colored urine, anorexia, jaundice of the skin, and loss of appetite, accompanied by clay-like loose stools. The liver function test conducted at the local hospital indicated the presence of liver failure, prompting the administration of intravenous drip therapy. However, despite this intervention, notable improvement in the patient’s condition was not observed. On June 11, 2021, the patient was brought to the emergency department of the First Affiliated Hospital of Kunming Medical University in order to receive medical care.

### 2.3. Past medical history

The patient did not have a medical history, including diabetes, cardiovascular and cerebrovascular diseases, lung diseases, malnutrition, endocrine diseases, and infectious diseases. There was no documented evidence of any injuries, surgery, blood transfusions, or allergies to drugs in his medical history. Additionally, the information regarding his vaccination history was not known.

### 2.4. Physical examination

The patient’s vital signs exhibited a body temperature of 36.7°C, a pulse of 99 beats per minute, a respiratory rate of 21 breaths per minute, and a blood pressure of 96/65 mm Hg. He was conscious, generally in poor condition, cooperative during physical examination. Additionally, he displayed jaundiced skin and sclera, with no observable enlargement of superficial lymph nodes across the body. The head and face showed no anomalous morphology, and both eyes displayed typical bilateral characteristics. There was no observed enlargement of the thyroid gland. During chest auscultation, it was seen that there was thoracic deformity, and the breath sounds emanating from both lungs were clear, with the absence of dry and moist rales. Cardiac examination was unremarkable with no observed enlargement of the heart border. The heart rate was measured at 99 beats/min, exhibiting regular rhythm, and no pathological murmurs were auscultated in the valves. Abdomen was flat with no tenderness or varicose veins observed in the abdominal wall. There was no visible intestinal pattern and peristaltic waves. However, rebound tenseness or muscular tenseness was present. The liver and spleen were not palpable below the costal arch. The result of Murphy sign was found to be negative. There was no percussion pain in the liver and kidney regions with no shifting dull sounds, and bowel sounds at 4 times per minute. No edema was observed in either lower extremity, neurophysiological responses were detected, and no pathogenic symptoms were evoked.

### 2.5. Laboratory data

His blood routine test conducted on June 11, 2021, revealed the following results: white blood cell count, 4.79 × 10^9^/L; neutrophils, 78.3%; lymphocytes, 9.9%; red blood cell count, 4.79 × 10^12^/L; hemoglobin, 147g/L; platelet count, 138 × 10^9^/L; plateletcrit, <0.05 mg/L; and C-reactive protein, 26.5pg/L. The liver tests demonstrated that alanine aminotransferase (ALT), 1793 U/L; aspartate aminotransferase (AST), 908 U/L; total bile acid, 156.8 μMol/L; albumin, 33 g/L; total bilirubin (TBIL), 79.40 μMol/L; direct bilirubin (DBIL), 58.1 μMol/L; and indirect bilirubin (IBIL), 21.00 μMol/L. The results of the coagulation tests indicated the following values: prothrombin time, 34 seconds; international normalized ratio, 1.78; activated partial thromboplastin time, 43.8 seconds; thrombin time, 21.8 seconds; fibrinogen, 3.38 g/L; and D-dimer, 0.37 mg/L. His blood ammonia level was 88 μmol/L, and all 4 tests conducted for hepatitis B yielded negative results. Further tests for tuberculosis, systemic lupus erthematosus, antineutrophil cytoplasmic antibody, rheumatoid related antibody, autoimmune hepatitis, anticardiolipin antibody, cytomegalovirus DNA, Epstein-Barr virus DNA, and novel coronavirus nucleic acid were negative.

### 2.6. Medical imagology

**Figure 1.**
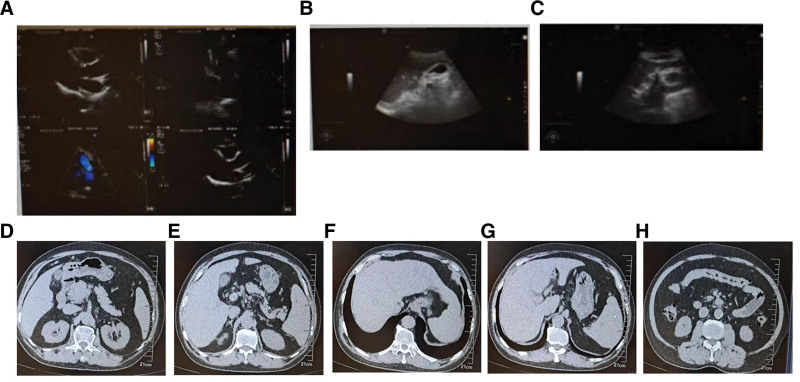
Changes in echocardiography, abdominal ultrasound, and abdominal MRI at admission. (A) Echocardiography; (B,C) abdominal ultrasound; (D–H) abdominal MRI. MRI = magnetic resonance imaging.

Abdominal ultrasound showed rough sonography of the left hepatic vein wall, thickened and coarse gallbladder wall, polypoid lesions of the gallbladder with a modest enlarged spleen (Fig. 1B,C). The echocardiography examination showed thickening of the interventricular septum and an increase in the diameter of the aorta (Fig. 1A). The abdominal MRI examination revealed that the liver parenchyma displayed a uniform signal, and no localized abnormal signals were observed. The liver displayed a normal size and shape, with appropriate proportions of each lobe. The liver parenchyma displayed a uniform signal, and no localized abnormal signals were observed. The spleen appeared slightly enlarged, while the intrahepatic and extrahepatic bile ducts, as well as the common bile duct, did not show any signs of enlargement. Furthermore, the gallbladder did not exhibit any enlargement (Fig. 1D–H).

### 2.7. Diagnosis and treatment

Based on an analysis of the patient’s medical history, symptoms, physical examination, and liver function tests, potential diagnoses of autoimmune hepatitis, systemic immune liver injury, and liver injury caused by cytomegalovirus and Epstein-Barr virus have been ruled out. The patient was diagnosed with live failure resulting from the use of ibuprofen-sustained release capsules. The patient initially ceased taking any of hepatotoxic drugs as a precautionary measure to mitigate further exposure. Subsequently, the patient was administered a daily dosage of 2 g of reduced glutathione and 150 mg of magnesium isoglycyrrhizinate for the purpose of liver protection. At the same time, nutritional supplement was given to correct acid-base balance and electrolyte disorders. During the course of treatment, the liver function indexes and the patient’s status were continuously monitored. On the third day of treatment, the liver function test revealed a significant decrease in the levels of ALT, AST, TBIL, and IBIL (Table 1). After continued treatment for 7 days, the patient’s symptoms were significantly improved, as seen by the resolution of jaundice in the skin and sclera, continued decrease of ALT and AST, and TBIL, DBIL, and IBIL returned to normal(Table 1). Following an assessment by the attending physician, the patient was discharged with prescribed drugs. The patient was advised against the consumption of ibuprofen tablets or capsules.

**Table 1:** Changes of liver function index before and after treatment.

	At admission	3 d after treatment	6 d after treatment	9 d after treatment	2 wks after discharge
ALT (U/L)	1793	1143	478	107	57
AST (U/L)	908	889	207	204.7	63
TBIL (μmol/L)	79. 40	81.8	32.4	31.2	26.4
DBIL (μmol/L)	58.1	60.6	27.8	24.9	19.8
IBIL (μmol/L)	25.4	21.9	4.6	6.3	6.6

ALT = alanine aminotransferase, AST = aspartate aminotransferase, DBIL = direct bilirubin, IBIL = indirect bilirubin, TBIL = total bilirubin.

### 2.8. Follow-up after treatment

The patient was followed up 2 weeks after discharge and did not have any discomfort. The liver function tests revealed that the levels of ALT and AST returned to normal, and the levels of TBIL, TBIL, and IBIL were within the normal range (Table 1).

## 3. Discussion

The incidence of DILI has shown a consistent upward trend in recent years.^[8]^ The prevalence of DILI in developed countries is rather modest, with the majority of drugs exhibiting a liver injury rate of (1–20)/100,000.^[9]^ Common causes of DILI in developed countries in Europe and America include nonsteroidal antiinflammatory drugs (NSAIDs), antiinfectious drugs, and herbal and dietary supplements. According to the data provided by the DILI Collaborative Network (DILIN), it is observed that herbal and dietary supplements accounts for >20% of the causes of DILl. Relevant literature in China shows that the top 5 categories of drugs for DILI are traditional Chinese medicine, antibiotics, antipyretic and analgesic drugs, antituberculosis drugs, and cardiovascular drugs.^[10]^ Therefore, NSAIDs are hepatotoxic and have the potential to induce liver injury. However, the precise mechanisms underlying liver damage induced by various NSAID subclasses remain little elucidated. Previous studies have shown that the mechanisms of liver injury induced by aniline, ibuprofen, and sodium diclofenac may be closely related to mitochondrial permeability transition, mitochondrial reactive oxygen species, mitochondrial Ca2+, and mitochondrial redox status.^[11]^ N-acetyl-P-benzoquinone imine (NAPQI) is produced by hepatic metabolism and possesses the ability to react with free thiols. Under normal circumstances, the hepatic detoxification process involves the rapid conjugation of NAPQI with glutathione. Excessive consumption of acetaminophen has been associated with the potential for inducing liver damage.^[12]^ Excess NAPQI can be covalently bound to free thiols, resulting in mitochondrial dysfunction, reactive oxygen species production, and cell mitogen activated protein kinase activation, thereby causing cell necrosis.^[13]^

NSAIDs are widely used in clinic and have a long history of use. The pharmacological action of NSAIDs mainly inhibits the synthesis of cyclooxygenase (COX) to block the synthesis of arachidonic acid into inflammatory mediators, namely prostaglandins, prostacyclins, thromboxanes, and leukotrienes, thereby exerting exert analgesic and antiinflammatory effects. The pathway is significantly influenced by 2 isoforms of COX, namely COX-1 and COX-2.^[14]^ Aspirin was first produced, followed by traditional NSAIDs and specific inhibitors of COX-2.^[14]^ NSAIDs are commonly classified into 3 distinct categories: (1) salicylate: aspirin, is the primary chemical within the salicylate class, (2) nonselective COX inhibitors: ibuprofen, indomethacin, naproxen, and diclofenac are classified within this group. (3) COX-2 inhibitors: celecoxib and nimesulide are categorized as members of this particular class. Therefore, the ibuprofen-sustained release capsule that the patient took here is a nonselective COX inhibitor. Ibuprofen is a nonspecific inhibitor of COX-1 and COX-2. Upon administration, it undergoes systemic absorption into the bloodstream. The majority of ibuprofen, exceeding 90.0%, binds to proteins and its primary metabolic processes occur within the liver. Ibuprofen is frequently employed as an analgesic agent, particularly for the management of pain associated with conditions such as rheumatism and is commonly utilized to alleviate symptoms such as fever, headache, toothache, and dysmenorrhea. Ibuprofen, an over-the-counter drug, is extensively utilized. It can cause adverse side effects such as peptic ulcer and gastrointestinal bleeding. It is generally believed that ibuprofen, as compared with other frequently utilized NSAIDs, exhibits a relatively lower propensity for inducing adverse hepatic effects and demonstrates a heightened level of safety in relation to liver function. However, there have been reported cases of patients experiencing fever, erythema multiforme, and jaundice following short-term ibuprofen administration. Drug lymphocyte stimulation test confirmed that the occurrence of liver injury associated with ibuprofen was due to idiosyncratic constitution.^[15]^ Studies have shown that concurrent administration of ibuprofen and aspirin can lead to a notable escalation in the incidence of liver toxicity and injury, particularly among individuals who consume alcohol.^[16]^ In this study, we found that ibuprofen-sustained release capsules can also cause liver failure.

At present, there exists no specific therapeutic intervention for DILI, and the treatment mainly involves detoxification and liver protection, and artificial liver treatment for patients with severe conditions. Reduced glutathione has been used in the treatment of DILI with certain effectiveness. Magnesium isoglycyrrhizinate is the fourth generation preparation of glycyrrhizic acid, which has been approved by Food and Drug Administration of China for its use in the treatment of acute DILI. It can be used to treat acute hepatocellular or mixed DILI with significantly elevated ALT, and can effectively reduce inflammatory cell infiltration, liver cell degeneration, and necrosis.^[17]^ Therefore, the patient received treatment involving the administration of reduced glutathione and magnesium isoglycyrrhizinate, resulting in successful detoxification and liver protection.

### 3.1. Strengths and limitations

#### 3.1.1. Strengths.

This case confirms that ibuprofen-sustained release capsules can cause liver failure, and treatment with detoxification, liver protection, and nutritional support is effective.

#### 3.1.2. Limitations.

The incidence of such cases is limited, and the underlying mechanisms leading to this condition remain uncertain. Further investigation is required to validate the impact of ibuprofen-sustained release capsules on hepatic function and elucidate its underlying mechanisms through animal studies and clinical trials. The efficacy of the treatment including the administration of reduced glutathione and magnesium isoglycyrrhizinate has been demonstrated. Due to the small number of cases, it needs to be further confirmed by larger sample size, multicenter, randomized controlled clinical trials.

## 4. Conclusion

In summary, there have been no clinical reports of liver failure caused by ibuprofen-sustained release capsules. This article introduces and analyzes the etiology, pathogenesis, diagnosis and treatment of liver failure caused by ibuprofen-sustained release capsules for the first time, so as to enhance healthcare professionals’ comprehension of the underlying mechanisms, therapeutic approaches and prognosis of such patients. Liver function tests should be conducted in cases when patients exhibit symptoms such as oil avoidance, skin jaundice, poor appetite, and nausea after taking ibuprofen-sustained release capsules. If liver injury occurs due to ibuprofen-sustained release capsules, it is important to promptly initiate appropriate medical intervention.

## Acknowledgments

This work was supported by the Nature Science Foundation of China under Grant [NO. 81960350]; Yunnan Applied Basic Research Project-Union Foundation of China under Grant [NO. 202201AY070001-091]; Yunnan Provincial Local University Joint Special Project under Grant [NO. 202001BA070001-141]; Lincang Innovative Talent Project under Grant [NO. 202304AC100001-RC14].

## Author contributions

**Conceptualization:** Ming-wei Liu.

**Data curation:** Yan Liu, Ming-wei Liu.

**Funding acquisition:** Ming-wei Liu.

**Investigation:** Yan Liu, Ming-wei Liu.

**Resources:** Yan Liu.

**Software:** Yan Liu.

**Supervision:** Yan Liu, Ming-wei Liu.

**Validation:** Yan Liu.

**Visualization:** Yan Liu.

**Writing – original draft:** Ming-wei Liu.

**Writing – review & editing:** Ming-wei Liu.
